# Biobased Postharvest Treatment Using Eucalyptus Essential Oils in Edible Coatings to Inhibit *Colletotrichum acutatum* and Prolong Strawberry Shelf Life

**DOI:** 10.3390/plants14162565

**Published:** 2025-08-18

**Authors:** Paula Porrelli Moreira da Silva, Nataly Maria Viva de Toledo, Jacqueline de Oliveira, Eduardo Micotti da Gloria, Fabiane Barco Maximo, Marta Helena Fillet Spoto

**Affiliations:** 1 Department of Food Science and Technology, “Luiz de Queiroz” College of Agriculture, University of Sao Paulo, Pádua Dias Av. 11, Piracicaba 13418-900, SP, Brazil; jacqueline.oliveira@alumni.usp.br (J.d.O.); fabiane.bmaximo@gmail.com (F.B.M.); martaspoto@usp.br (M.H.F.S.); 2 Piracicaba College of Technology (FATEC) Diácono Jair de Oliveira, Undergraduate Program in Food Science, Av. 651, Piracicaba 13414-155, SP, Brazil; 3 Biological Sciences Department, “Luiz de Queiroz” College of Agriculture, University of Sao Paulo, Pádua Dias Av. 11, Piracicaba 13418-900, SP, Brazil; emgloria@usp.br

**Keywords:** natural antifungal, carboxymethylcellulose, sensory characteristics, in vitro assay, in vivo assay

## Abstract

Strawberries are economically valuable but highly perishable fruits, mainly due to fungal spoilage, with no fungicides currently registered for postharvest use. This study aimed to develop a biobased postharvest treatment for strawberries focusing on fungal control and shelf-life extension. The antifungal activity of *Eucalyptus staigeriana*, *Eucalyptus urograndis*, and their binary mixture was evaluated in vitro against the spoilage fungus *Colletotrichum acutatum*. The effects on pathogen morphology, in vivo efficacy when incorporated into carboxymethylcellulose (CMC), and impacts on postharvest and sensory quality of strawberries were also assessed. *E. staigeriana* EO showed the highest antifungal activity in vitro. In vivo, the incorporation of *E. staigeriana* EO into CMC significantly reduced disease severity when applied curatively. Treated fruits exhibited less fungal decay during refrigerated storage, indicating improved preservation. However, sensory evaluation revealed changes mainly in the aroma of the fruit. These results suggest that *E. staigeriana* EO combined with CMC coating is a promising postharvest antifungal treatment for strawberries, though further research is needed to optimize the formulation and reduce sensory impacts.

## 1. Introduction

Although strawberries (*Fragaria* × *ananassa*) are recognized for their high commercial value, their extreme perishability poses a major challenge throughout the production chain. One of the main factors limiting their postharvest shelf life is their susceptibility to phytopathogenic fungi, such as *Botrytis cinerea*, *Colletotrichum acutatum*, and *Rhizopus stolonifer* [1,2], which rapidly deteriorate fruit quality and result in substantial economic losses.

Traditionally, synthetic fungicides and chemical substances have been extensively used to control fungal spoilage in strawberries. These methods are generally effective but raise concerns regarding chemical residues, environmental impact, and the development of resistant fungal strains [3,4]. Physical methods, such as controlled atmosphere storage and refrigeration, are also commonly applied to extend shelf life, though they may not fully prevent fungal growth.

In recent years, alternative natural postharvest strategies have gained attention as safer and more sustainable options, including microbial antagonists, microbial fermentates, nano-formulated delivery systems, plant-derived extracts, and other biobased coatings, which have shown promising results in controlling spoilage fungi and maintaining fruit quality [5,6,7]. In countries like Brazil, where no fungicides are currently registered for postharvest application on strawberries, such alternatives are particularly relevant.

Within this context, essential oils (EOs) from aromatic plants stand out for their broad-spectrum antimicrobial activity and potential for effective pathogen control [8]. Among them, EOs derived from eucalyptus species (e.g., *Eucalyptus staigeriana* and *Eucalyptus urograndis*) are especially notable due to their richness in bioactive compounds, which have shown efficacy against spoilage fungi in different crops [9,10,11].

However, the direct application of EOs to fresh produce is known to cause undesirable effects, including negative sensory impacts and damage to the structural integrity of the food. As a result, technologies such as incorporation into edible coatings or microencapsulation have proven effective in protecting volatile compounds while enabling the controlled and targeted release of active substances [8,12,13]. Despite the potential of these strategies, studies evaluating EO application via edible coatings in conjunction with postharvest fruit quality assessment remain scarce, particularly regarding EOs from *E. staigeriana* and, notably, *E. urograndis*, for which evidence of antifungal efficacy against *C. acutatum* is virtually nonexistent. Current data are largely limited to antibacterial activity against certain strains of *Bacillus* and *Streptococcus* [9,14], as well as antifungal activity against *Rhizopus stolonifer* and *Botrytis cinerea* [15].

This study is therefore significant as it investigates not only the antifungal activity of these EOs but also their effects on pathogen morphology and the physicochemical and sensory quality of strawberries. Specifically, the aim was to evaluate whether the EOs of *E. staigeriana*, *E. urograndis*, and their binary mixture, combined with carboxymethylcellulose (CMC), exhibit in vitro and in vivo antifungal activity against *C. acutatum* isolated from strawberries. In addition, the EO showing the greatest antifungal efficacy was applied, in combination with CMC, to strawberries in order to assess its impact on postharvest quality and sensory attributes during refrigerated storage. The promising results of this research may serve as a foundation for future studies exploring the application of these essential oils in other plant-based food matrices.

## 2. Results

### 2.1. In Vitro Antifungal Activity of the EOs Against Colletotrichum acutatum Isolated from Strawberries

**Figure 1:**
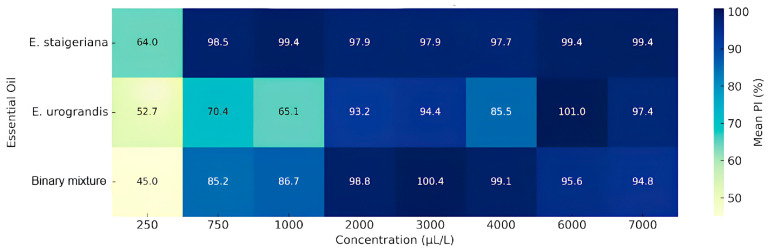
Heat map representing the mean percentage of *C. acutatum* growth inhibition (PI%) as a function of different concentrations (µL L^−1^) of *E. staigeriana* and *E. urograndis* essential oils and their binary mixture (50:50). Color intensity indicates the degree of inhibition, with darker shades representing greater antifungal efficacy.

The essential oils and the binary mixture inhibited the growth of *Colletotrichum acutatum* in a concentration-dependent manner. The *Eucalyptus staigeriana* EO was the most effective, showing significant inhibition from 750 µL L^−1^ (PI > 97%), while *Eucalyptus urograndis* and the binary mixture showed greater efficacy from 2000 µL L^−1^, with PI values above 93% and 98%, respectively (Table 1). These results are supported by the heat map (Figure 1) and by the MIC and MFC values, highlighting the superior performance of *E. staigeriana* EO (MIC > 750 µL L^−1^; MFC > 2000 µL L^−1^), even at lower concentrations. The binary mixture exhibited intermediate performance (MIC > 2000 µL L^−1^; MFC > 4000 µL L^−1^), whereas the *E. urograndis* EO required the highest concentrations for both inhibition and fungicidal effect (MIC > 6000 µL L^−1^; MFC 7000 µL L^−1^), indicating unsatisfactory activity.

EC_50_ is the concentration of a compound required to cause 50% inhibition of fungal mycelial growth compared to the control under in vitro experimental conditions. Accordingly, the *E. staigeriana* EO showed the lowest EC_50_ value (185.49 µL L^−1^), while the *E. urograndis* EO and the binary mixture required higher concentrations (337.01 and 355.62 µL L^−1^, respectively) to inhibit 50% of fungal growth, confirming the superior efficacy of *E. staigeriana* in suppressing the development of *C. acutatum* (Table 2).

For comparison purposes, the effects of *E. staigeriana* and *E. urograndis* essential oils on the morphology of *C. acutatum* were evaluated, since the former exhibited the lowest MIC, MFC, and EC_50_ values, while the latter showed the highest.

### 2.2. Morphological Effect of Essential Oils from Eucalyptus staigeriana and Eucalyptus urograndis on the Fungus

Scanning Electron Microscopy (SEM) analysis also revealed antifungal effects of the essential oils on *C. acutatum*, showing structural alterations in the hyphae. In the absence of EOs, the hyphae exhibited a smooth, continuous, and intact surface, with elongated morphology and preserved structure.

**Figure 2:**
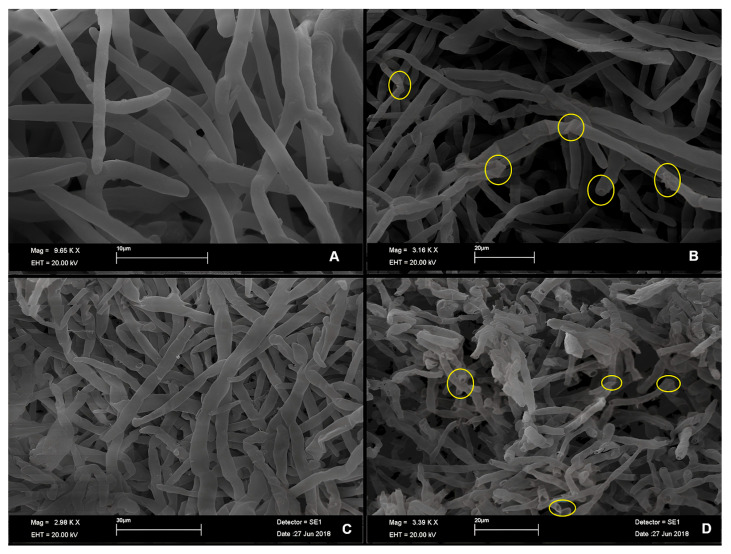
Effect of essential oils on the morphology of *C. acutatum* hyphae (strawberry isolate), observed by Scanning Electron Microscopy. (**A**,**C**): untreated hyphae (controls). (**B**): hyphae after 6 h of exposure to 2000 µL L^−1^ of *Eucalyptus staigeriana* EO. (**D**): hyphae after 6 h of exposure to 4000 µL L^−1^ of *Eucalyptus urograndis* EO. Circles indicate major areas of structural damage caused by the essential oils.

Samples treated with *E. staigeriana* EO (2000 µL L^−1^) showed deformations such as cell wall roughness, collapse, and loss of regular morphology, with regions exhibiting twisting of hyphae (Figure 2A,B). Meanwhile, *E. urograndis* EO (4000 µL L^−1^) caused less severe alterations, including surface irregularities, flattening, distortion, and loss of linearity (Figure 2C,D). These results are consistent with the in vitro assay data, which demonstrated greater efficacy of *E. staigeriana* EO, even at lower concentrations.

Therefore, for the in vivo and postharvest experiments, the *E. staigeriana* EO was selected due to its higher antifungal efficacy in previous tests (lower MIC, MFC, and EC_50_ values).

### 2.3. In Vivo Antifungal Activity of Essential Oils Incorporated into a Carboxymethylcellulose Edible Coating Against Colletotrichum acutatum

**Figure 3:**
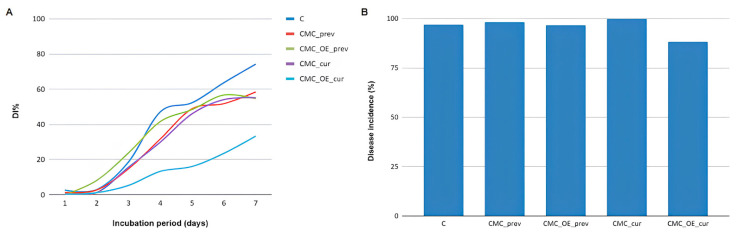
Disease progression curves represented by the disease index (DI%) (**A**), and average disease incidence (%) (**B**) in symptomatic strawberries inoculated with *C. acutatum* after 7 days of storage. Data correspond to the mean of two experiments conducted one year apart, under controlled incubation conditions (7 days, 95% RH, 25 °C, and 12 h photoperiod), with application of different treatments. C: fruits without application of carboxymethylcellulose or essential oil (control); CMC_prev: fruits treated preventively with carboxymethylcellulose only; CMC_OE_prev: fruits treated preventively with carboxymethylcellulose and essential oil; CMC_cur: fruits treated curatively with carboxymethylcellulose only; and CMC_OE_cur: fruits treated curatively with carboxymethylcellulose and essential oil.

The disease index (DI%) represents the proportion of infected fruits throughout the incubation period. The combination of carboxymethylcellulose with *E. staigeriana* EO showed the lowest fungal development when applied after infection (CMC_OE_cur), indicating potential use of the curative treatment in postharvest situations with early symptoms (Figure 3A).

Regarding disease incidence (Figure 3B), considering the average of the two experimental repetitions, all treatments showed high values, close to 100%. This result was expected due to the high inoculum pressure and the use of ripe fruits, combined with incubation conditions favorable to the fungus (95% RH, 25 °C, 12 h photoperiod), which were deliberately set to stimulate its development.

The Area Under the Disease Progress Curve (AUDPC) quantitatively summarizes disease intensity over time by combining multiple observations of disease progression into a single value [16]. Thus, the lower the AUDPC value, the lower the disease severity over time, indicating a better treatment effect.

The disease severity caused by *C. acutatum*, represented by AUDPC values, shows that the curative treatment with *E. staigeriana* EO incorporated into CMC (CMC_EO_cur, 78.45) was the most effective in reducing the progression of infection over time, with a statistically significant difference (*p* < 0.05) compared to the other treatments (Table 3). Conversely, the preventive application of the EO (CMC_EO_prev, 223.13) did not differ from the control, indicating that the curative use of the essential oil is more promising for controlling *C. acutatum* under postharvest conditions, even when applied after infection onset.

These results reinforce the potential of *E. staigeriana* EO as an antifungal agent and its application with CMC as a viable strategy to extend the shelf life of strawberries.

### 2.4. Postharvest and Sensory Parameters of Strawberries Treated with CMC Incorporated with EO of Eucalyptus staigeriana

**Figure 4:**
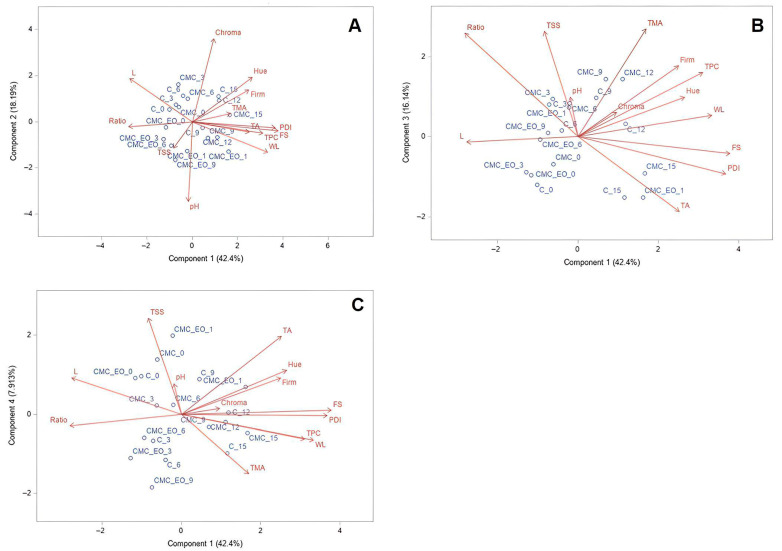
Distribution plots of variables and observations from Principal Component Analysis of postharvest parameters in strawberries treated or untreated with *E. staigeriana* essential oil, stored under refrigeration for 15 days. (**A**) = Principal Component 1 vs. Principal Component 2; (**B**) = Principal Component 1 vs. Principal Component 3; (**C**) = Principal Component 1 vs. Principal Component 4. Variables: PDI = pericarp disease incidence index; FS = fungal spoilage; WL = fresh weight loss; L = lightness; Chroma = chroma; Hue = hue angle; TSS = total soluble solid; pH = pH; Firm = firmness; TA = titratable acidity; Ratio = palatability index; TMA = total monomeric anthocyanin; and TPC = total phenolic compound. Observations (treatments_storage periods): C = control strawberries; CMC = strawberries treated with carboxymethylcellulose (CMC); CMC_OE = strawberries treated with CMC incorporated with *E. staigeriana* essential oil; and refrigerated storage periods (days): 0, 3, 6, 9, 12, and 15.

Principal Component Analysis (PCA) extracted four main components from the full dataset (Table 4), accounting for 84.64% of the total variance (Figure 4). The first principal component (PC1) explained 42.40% of the variance and was positively correlated with fungal spoilage (FS), pericarp disease incidence (PDI), weight loss (WL), and total phenolic compounds (TPCs) while showing a negative correlation with the ratio (RT).

The second principal component (PC2) accounted for 18.19% of the variance and was positively correlated with chroma and negatively correlated with pH. The third principal component (PC3) explained 16.14% of the variance and was positively associated with total monomeric anthocyanins (TMAs) and total soluble solids (TSSs). The fourth principal component (PC4) accounted for 7.91% of the variance and was positively correlated with titratable acidity (TA).

The variables lightness, firmness, and hue angle were not significantly associated with any principal components, as they did not contribute relevant information in the analysis.

Strawberries treated with *E. staigeriana* EO and stored under refrigeration for up to 12 days (CMC_EO_0, CMC_EO_3, CMC_EO_6, CMC_EO_9, and CMC_EO_12) showed lower values of fungal spoilage and disease index compared to the other treatments (control and CMC), indicating that the use of EO can positively contribute to the quality and shelf life of strawberries. This result was expected, given that a similar antifungal effect of *E. staigeriana* EO was observed in both the in vitro and in vivo evaluations conducted in this study. Furthermore, the application of *E. staigeriana* EO to the strawberries can be likened to the curative treatment used in the in vivo assay, considering that the fruits were likely contaminated in the field (prior to harvest and treatment). In that assay, the curative application yielded the most effective results against *C. acutatum*. It is also noteworthy that all treatments showed increasing values for both fungal spoilage and disease index throughout the storage period.

Fruits treated with CMC_EO and stored under refrigeration for up to 12 days (observations CMC_EO_0, CMC_EO_3, CMC_EO_6, CMC_EO_9, and CMC_EO_12) also exhibited lower weight loss compared to the CMC treatment. However, these samples presented lower levels of total phenolic compounds compared to the control samples. Regarding the ratio, the application of *E. staigeriana* EO to strawberries was beneficial, as samples treated with the essential oil and stored for up to 9 days (CMC_EO_0, CMC_EO_3, CMC_EO_6, and CMC_EO_9) showed higher values compared to the other treatments.

**Figure 5:**
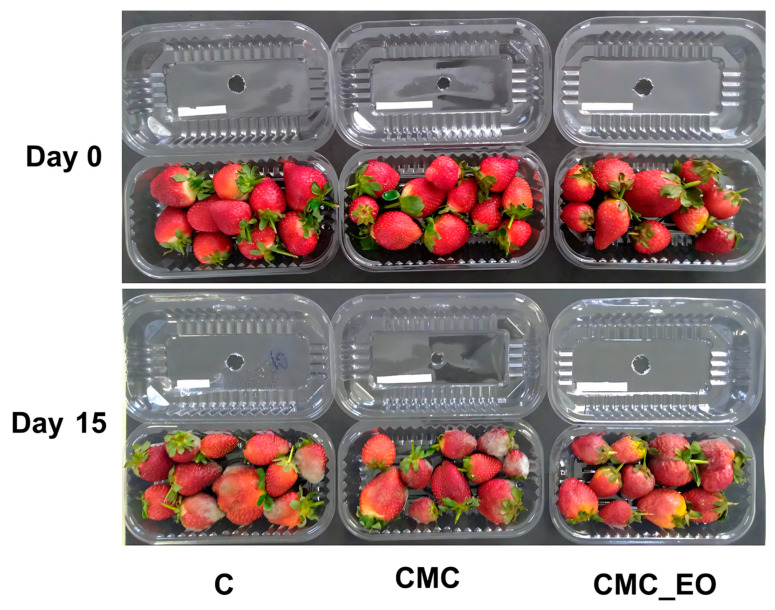
Appearance of strawberries artificially inoculated with *C. acutatum* and either untreated or treated with carboxymethylcellulose incorporating *E. staigeriana* essential oil, shown immediately after processing (Day 0) and after 15 days of refrigerated storage (Day 15). C = control strawberries; CMC = strawberries treated with carboxymethylcellulose; and CMC_EO = strawberries treated with carboxymethylcellulose combined with *E. staigeriana* essential oil. Each treatment was performed in triplicate, with each replicate consisting of 150 g of fruit; the images shown correspond to one representative replicate per treatment.

Among the color parameters, chroma (color saturation) was represented in Principal Component 2. It was also observed that strawberries treated with *E. staigeriana* EO and stored for up to 12 days under refrigeration exhibited lower color saturation, indicating a less pure or vivid color (Figure 4). Although the hue angle parameter was not represented in the displayed principal components, it is important to note that the strawberries maintained a red color throughout the storage period (Figure 5).

The Difference from Control Test was used to compare treated samples, with or without *E. staigeriana* essential oil (CMC_EO and CMC, respectively), to the control regarding characteristic color, characteristic aroma, appearance, and overall sensory difference. The results demonstrate that both the CMC coating and the addition of *E. staigeriana* EO induced noticeable sensory changes compared to the control strawberries. The presence of the essential oil intensified these changes across all attributes, with a significant difference (*p* < 0.05) observed in characteristic aroma when compared to the control (Table 5). The absence of a significant difference in appearance between the CMC treatment and the control suggests that the coating alone does not compromise the product’s visual aspect, which may positively influence consumer acceptance.

These findings indicate that, overall, the addition of CMC and *E. staigeriana* EO altered the sensory characteristics of the strawberries, with characteristic aroma being the most affected attribute. In this context, the descriptors most frequently reported by panelists regarding the aroma of the EO-treated samples included: cleaning product, eucalyptus, lemon balm, menthol, citronella, synthetic, citrus, disinfectant, and clove.

The applied concentration of *E. staigeriana* EO (6000 µL L^−1^) was considered high, which may have negatively influenced the fruit’s sensory properties. Consequently, the control sample was preferred by 55.26% of the panelists, while only 8% chose the EO-treated sample. These results suggest that, although the use of *E. staigeriana* EO can add functional properties to the product, its application in strawberries requires adjustment of concentration or incorporation method to minimize undesirable sensory impacts and improve consumer acceptance.

## 3. Discussion

This study is the first to evaluate the effects of essential oils from *Eucalyptus staigeriana* and *Eucalyptus urograndis* on *Colletotrichum acutatum* isolated from strawberries, encompassing in vitro and in vivo tests, as well as the application of *E. staigeriana* EO incorporated into CMC for postharvest preservation and sensory quality assessment. *E. staigeriana* EO demonstrated the greatest potential for anthracnose control while maintaining strawberry quality during refrigerated storage. The effectiveness of EOs against different pathogens is associated with the diversity of phytochemical compounds, which, through distinct modes of action, lead to cell death [17,18].

In the in vitro assays, all tested EOs inhibited the growth of *C. acutatum* in a concentration-dependent manner. *E. staigeriana* EO showed the best performance, with an MIC of 750 µL L^−1^ and an MFC of 2000 µL L^−1^, whereas *E. urograndis* EO was the least effective, requiring much higher concentrations (MIC >6000 µL L^−1^; MFC 7000 µL L^−1^). The binary mixture showed an intermediate effect (MIC >2000 µL L^−1^; MFC >4000 µL L^−1^), possibly due to the dilution of active compounds from *E. staigeriana* or interference between constituents of both eucalyptus species, which may have limited the expected synergistic effect. This lack of a synergistic effect is not uncommon in the literature. While some studies have reported synergistic interactions, such as Nikkhah et al. (2017) [19], who observed synergy between thyme and cinnamon oils against *Penicillium expansum* and *B. cinerea* on pear fruit, other research has shown that combining cinnamaldehyde and clove essential oils can produce indifferent or antagonistic effects, both in vitro and in vivo (tomato fruit system), against various fungal species (*Aspergillus*, *Fusarium*, *Penicillium*, and *Rhizopus*) [20]. This suggests that complex interactions, and not necessarily synergy, are a common outcome when combining different essential oils and should be considered in future formulations.

The EC_50_ values confirmed this trend: *E. staigeriana* (185.49 µL L^−1^) < *E. urograndis* (337.01 µL L^−1^) < binary mixture (355.62 µL L^−1^).

*E. staigeriana* EO is mainly composed of limonene (14.93%), α-thujone (11.22%), and geranial (8.34%), while *E. urograndis* EO contains 1,8-cineole (eucalyptol, 41.34%), α-pinene (27.66%), and α-terpinyl acetate (7.95%) [15]. Although both are rich in terpenoids, antifungal activity depends not only on the presence of individual compounds but also on their interactions. Synergistic or antagonistic effects may occur, making it difficult to attribute the activity to a single constituent [21].

Previous studies support the findings observed in this work. Pedrotti et al. (2020) [22] obtained *E. staigeriana* essential oil with a chemical composition similar to that used in the present study, predominantly composed of citral (30.91%), 1,8-cineole (24.59%), and limonene (19.47%). This EO was effective in inhibiting both mycelial growth and conidial germination of *C. acutatum* isolated from grapes, showing significant antifungal activity starting at 500 µL L^−1^ in in vitro assays. Later, Pedrotti et al. (2022) [23] demonstrated that the same EO was capable of controlling *Colletotrichum gloeosporioides* and *Greeneria uvicola* in grapes, with complete inhibition of conidial germination at 500 µL L^−1^ and of mycelial growth at 1000 µL L^−1^. Furthermore, *E. staigeriana* EO completely inhibited both the mycelial growth and conidial germination of *Alternaria alternata* at 1000 µL L^−1^ (MIC and MFC), with significant reductions also observed at lower concentrations (250 and 500 µL L^−1^) [24].

Among the major compounds present in *E. staigeriana* EO, α-thujone has demonstrated strong antifungal activity against *Fusarium graminearum*, a phytopathogen that affects cereals and causes severe morphological, genetic, epigenetic, and cellular alterations [25]. Geranial, another key compound, acts by disrupting microbial cell membranes, leading to cell lysis and death, thereby contributing to the reduction in microbial load in food products [26,27].

The EO of *E. urograndis* remains poorly studied, particularly in assays against *C. acutatum*. Borges et al. (2024) [28] identified a high proportion of oxygenated monoterpenes (69%) and oxygenated sesquiterpenes (22%) in its composition, with notable compounds including 1,8-cineole, α-terpineol, α-terpinyl acetate, and caryophyllene oxide. Maronde et al. (2020) [29] also identified 1,8-cineole (54.76%) and α-pinene (22.72%) as the main components of this EO. Zhang et al. (2022) [30] demonstrated that 1,8-cineole disrupts biofilm formation and adhesion of *Fusarium solani*, affecting membrane organization, inhibiting hyphal formation and adhesion, reducing extracellular matrix synthesis, and impairing mitochondrial activity. α-Pinene has also shown antifungal activity against *Saccharomyces cerevisiae* cells, primarily through membrane damage that leads to ion leakage and altered electrical conductivity [31].

Moreover, comparative studies have shown that limonene, the major compound in *E. staigeriana* EO, exhibited fungistatic activity against 33.3% of Candida strains tested, whereas eucalyptol (1,8-cineole), the dominant compound in *E. urograndis* EO, produced no inhibition zones in the same disk diffusion assays [32]. Although these assays were conducted on human pathogenic microorganisms, the findings support the results of this study, in which the limonene-rich EO demonstrated greater antifungal efficacy. Overall, limonene is more extensively studied and consistently presents antifungal properties, while evidence of the antifungal action of eucalyptol remains more limited and less frequent in direct comparative studies [33,34].

A critical comparison of our findings with the existing literature reveals a wide range of antifungal efficacy for essential oils against *Colletotrichum* species, underscoring the influence of both chemical composition and experimental conditions. For example, *Myrcia ovata*, a chemotype combining nerolic acid and linalool, exhibited MIC and MFC values of 30 µL L^−1^ and 100 µL L^−1^, respectively [35], while cinnamon oil showed MIC/MFC values of 200 µL L^−1^ against the fungus isolated from ‘Hongyang’ kiwifruit [36]. In a study involving *C. acutatum* isolated from strawberries, Morkeliūnė et al. (2021) [37] found that thyme essential oil completely inhibited mycelial growth at concentrations above 200 µL L^−1^. In comparison, the MIC and MFC values obtained for *E. staigeriana* EO in the present study were approximately 7 to 10 times higher, indicating that it was less potent against the tested fungus.

The superior efficacy of these oils compared to *E. staigeriana* EO may be attributed to differences in their major active compounds and their concentrations, which often influence the mode of action. This highlights the need for a targeted approach in selecting essential oils for postharvest applications.

Studies on the EC_50_ values of EOs against *C. acutatum* remain limited. Apiole, a major constituent of parsley (*Petroselinum crispum*) essential oil, showed an EC_50_ of 40 µL L^−1^ against *C. acutatum* [38], which is lower than the values observed in the present study (*E. staigeriana*: 185.49 µL L^−1^; *E. urograndis*: 337.01 µL L^−1^). Conversely, EOs from lemon and sweet orange, as well as limonene, exhibited EC_50_ values between 5500 and 6540 µL L^−1^ against *Colletotrichum okinawense* [34]. This suggests that the efficacy of an essential oil is highly dependent on both the fungal species and the chemical composition of the oil itself. The lower EC_50_ values of the eucalyptus essential oils used in this study indicate a more potent effect compared to citrus oils, supporting their potential as viable alternatives for managing specific pathogens such as *C. acutatum*.

Morphological analysis using Scanning Electron Microscopy (SEM) revealed that *E. staigeriana* EO caused more severe damage to *C. acutatum* hyphae than *E. urograndis* EO, with structural alterations indicative of lysis and cellular collapse. These findings corroborate the in vitro results and support the selection of *E. staigeriana* EO for in vivo testing. Similar alterations have been reported by Oliveira et al. (2019) [39] using *Lippia sidoides* EO, and by Rashid et al. (2018) [40], who observed hyphal wrinkling, peeling, and lysis following treatment with *Rhus coriaria L.* extract. Likewise, SEM analyses have shown that the essential oils of *Origanum vulgare* and *Cymbopogon citratus* can inhibit growth and induce morphological changes in *Colletotrichum* spp. and other postharvest fungi [41].

In the in vivo stage of this study, a high incidence of anthracnose was observed across all treatments, which was expected due to the elevated inoculum pressure and favorable conditions for fungal development. This drop in efficacy from in vitro to in vivo conditions is a well-documented phenomenon in postharvest studies with essential oils. The antifungal efficacy often declines in vivo due to environmental complexity, physical and biological barriers, and the inherent volatility of the compounds [42]. This observation is supported by studies that have noted reduced effectiveness in vivo compared to in vitro results [20,43]. Nevertheless, only the curative treatment with *E. staigeriana* EO (CMC_EO_cur) was effective in reducing disease severity, presenting the lowest AUDPC value. In contrast, the preventive treatment did not show significant effectiveness, suggesting that its action is more efficient when applied after infection onset, an important consideration for postharvest disease management. This suppressive effect in curative mode had already been observed by our research group in a previous study [15], in which soft rot caused by *R. stolonifer* was reduced in strawberries treated with a similar combination of EO and CMC.

The lower efficacy observed in the preventive treatment may be related to the interaction between the CMC coating and the essential oil with the fruit matrix, which can reduce the oil’s availability before infection is established. In contrast, in the curative application, the treatment is administered after inoculation or the onset of infection, allowing the essential oil to act directly on active fungal structures (such as mycelium and spores) within the fruit tissue. Essential oils are often more effective when applied after infection has been established, as their volatile compounds are more likely to interact with vulnerable fungal structures, thereby disrupting critical physiological processes such as membrane integrity and mitochondrial respiration. This can result in the immediate interruption of fungal growth and lesion formation, as previously demonstrated for clove essential oil applied curatively against *Nigrospora* sp. in papayas [44]. Moura et al. (2024) [45] also emphasize the effectiveness of curative treatments, showing that the application of Pera IAC sweet orange essential oil at a concentration of 32,000 µL L^−1^ reduced the severity of sour rot caused by *Geotrichum citri-aurantii* in Tahiti acid limes by up to 96% when applied after infection onset.

Given the demonstrated efficacy of essential oils, particularly in the curative treatment against *C. acutatum*, their application in postharvest preservation systems emerges as a promising strategy. It is well known that edible coatings help extend the shelf life of fruits and reduce postharvest losses by minimizing water loss and slowing the respiration rate. When enriched with EOs, these coatings provide additional protection due to the broad-spectrum antimicrobial activity of essential oils, which are effective against a wide range of pathogens responsible for microbial spoilage and postharvest deterioration [46].

Among the bioactive compounds found in EOs, constituents such as limonene, α-thujene, and geranial, abundantly present in *E. staigeriana* EO [15], play significant roles in fruit ripening and defense mechanisms, primarily due to their antimicrobial, antioxidant, and physiological effects [47].

The application of eucalyptus EO has emerged as a natural, effective, and environmentally sustainable alternative for controlling fungal diseases. Studies have reported promising results regarding its impact on the postharvest quality of various fruits, including the use of *E. staigeriana* on grapes [22,23], *E. camaldulensis var. obtusa* on strawberries [48], and *E. globulus* on oranges [49], as well as on apples and pears [50].

In our study, the EO of *E. staigeriana* contributed to a reduction in fungal spoilage and disease index over 12 days of refrigerated storage. These results are consistent with those reported by Pedrotti et al. (2022) [23] in grapes, further highlighting the potential of this EO to improve postharvest quality across a variety of fruits.

Our findings are in line with those of Abd-Elkader et al. (2021) [48], who demonstrated that the application of *Eucalyptus camaldulensis* EO reduced weight loss and helped mitigate texture degradation in strawberries during refrigerated storage. Similarly, Adeogun et al. (2025) [49] reported positive outcomes using eucalyptus EO combined with carboxymethylcellulose in oranges, including reduced weight loss, enhanced antioxidant activity, and improved firmness retention, supporting the effectiveness of eucalyptus EO-based treatments in extending postharvest shelf life.

Strawberries are particularly susceptible to rapid water loss due to their thin, delicate skin, which can compromise appearance and firmness, which are critical visual attributes influencing consumer acceptance. This structural characteristic also presents specific challenges and opportunities for the application of essential oils, differing from those encountered in fruits with a thicker, waxy cuticle, such as apples or citrus. This highlights that a successful application in one matrix does not guarantee the same outcome in another, emphasizing the need for matrix-specific optimization for each fruit or vegetable.

Beyond its application in fresh produce, eucalyptus essential oil also shows promise as a natural food preservative in processed products, such as fruit juices. This is an attribute that is highly valued by both the food industry and consumers who seek cleaner label alternatives [51].

A key challenge for the commercial application of essential oils in postharvest coatings and food systems lies in their impact on sensory characteristics and consumer acceptance, which requires a critical analysis of their olfactory profile. Due to their complex chemical composition, essential oils are responsible for the distinctive aroma and flavor of various herbs and spices and may also act as functional ingredients with bioactive properties. Nonetheless, depending on the concentration and the food matrix, the sensory alterations they induce can limit their use [52].

Despite the promising results obtained in this study, the application of CMC and *E. staigeriana* EO led to changes in most sensory attributes of strawberries stored under refrigeration for 6 days. Such effects are consistent with the fact that the potential for sensory rejection often correlates with the intensity and specific profile of an essential oil’s aroma.

Previous studies indicate that the sensory acceptance of fruits treated with essential oils varies according to the concentration and type of oil used. For example, Pedrotti et al. (2022) [23] reported good acceptance of grapes treated with *E. staigeriana* EO at low concentrations, whereas Giello et al. (2024) [53] documented sensory rejection of more intense EOs, such as thyme oil, at higher concentrations. Conversely, Shehata et al. (2020) [54] reported that strawberries treated with citrus EOs exhibited greater acceptance and overall sensory quality than untreated fruits after storage, as the treatment enhanced freshness and flavor, leading to more favorable sensory attributes.

In addition to using lower EO concentrations, strategies such as combining antimicrobial agents at moderate doses may represent promising alternatives to mitigate the negative sensory effects associated with EO application in strawberries [55]. It is recommended that the concentration of essential oils penetrating the fruit pulp remain below the sensory threshold to achieve greater consumer acceptance [56].

Another suggestion for future studies with *E. staigeriana* EO is the application of advanced delivery systems, such as nanoemulsion-based edible coatings, which have shown promise as effective alternatives to mitigate the sensory effects associated with essential oil application while preserving their antimicrobial efficacy [57].

Therefore, these findings highlight the importance of optimizing essential oil concentrations and exploring alternative delivery systems to minimize sensory drawbacks. Such approaches are essential to increase consumer acceptance and support the commercial viability of essential oil-based technologies for the preservation of fresh produce.

## 4. Materials and Methods

### 4.1. Isolation, PCR-Based Identification of Colletotrichum acutatum, and Plant Materials

Isolates of *Colletotrichum acutatum* were obtained from fungal structures present in infected strawberries collected from a conventional farm in the municipality of Jarinu (São Paulo, Brazil). Fungal structures were cultured on Potato Dextrose Agar (PDA) medium and incubated at 25 °C under a 12 h photoperiod. Genomic DNA was extracted using the FastDNA^®^ Kit (MP Biomedicals, São Caetano do Sul, Brazil) and identified as belonging to the *C. acutatum* species complex through PCR amplification with the universal ITS4 primer and the species-complex-specific Cacut-Int2 primer. Amplification was confirmed by gel electrophoresis, identifying the pathogen as belonging to the *C. acutatum* species complex as described by Oliveira et al. (2019) [39].

The essential oils (EOs) from the leaves of *Eucalyptus staigeriana* and *Eucalyptus urograndis* were obtained by hydrodistillation, and their complete chemical composition was determined as previously described by our group in da Silva et al. (2020) [15]. To avoid redundancy, the detailed chemical profiles, including the identification and quantification of major components, are not presented. However, these data were used to support the discussion regarding the antifungal activity of the EOs in this study.

‘Oso Grande’ strawberries were harvested from an organic farm located in Cambuí (Minas Gerais, Brazil). Only fruits exhibiting uniform size and free from visible physiological damage or signs of microbial infection were selected. For both the in vivo assays and postharvest analyses, strawberries at the “fully ripe red” maturation stage were chosen. This stage is characterized by an even red coloration across the entire surface, along with full development of size and firmness. It was deliberately selected as it represents the ideal stage for commercialization while also being more susceptible to fungal infection, thus enabling clearer differentiation of treatment effects.

### 4.2. In Vitro Antifungal Activity of Essential Oils Against C.acutatum Isolated from Strawberries

The in vitro antifungal activity of the essential oils from *E. staigeriana* and *E. urograndis*, as well as their binary mixture (BM) composed of 50% of each EO, was evaluated using the following parameters: Percent Growth Inhibition (PI), Minimum Inhibitory Concentration (MIC), Minimum Fungicidal Concentration (MFC), and Median Effective Concentration (EC_50_). The analyses were carried out using the broth microdilution method, as described by da Silva et al. (2020) [15].

Accordingly, eight concentrations of each EO and the binary mixture (BM) were tested: 250, 750, 1000, 2000, 3000, 4000, 6000, and 7000 µL L^−1^, based on preliminary tests conducted on solid medium (PDA). As inoculum, a spore suspension in potato dextrose broth (PDB) containing 10^5^ spores mL^−1^ was used, obtained from *C. acutatum* colonies grown for 7 days on PDA.

The EOs and BM were initially emulsified in dimethyl sulfoxide (DMSO) at a 1:1 ratio (EO:DMSO) and diluted in PDB to obtain the stock solutions. Treatments were prepared in 96-well microplates, with a final volume of 200 μL per well, following these compositions: T1—150 μL of DMSO in PDB + 50 μL of spore suspension; T2—200 μL of DMSO in PDB; T3—200 μL of EO stock solution; and T4—150 μL of EO stock solution + 50 μL of spore suspension.

Each concentration was tested using three technical replicates (wells), and the entire assay was independently repeated three times on different days, totaling three biological replicates. After 48 h of incubation (25 °C, 12 h photoperiod), fungal growth was evaluated by measuring absorbance at 600 nm using a microplate reader (VictorX, Perkin Elmer, Waltham, MA, USA).

The percentage of fungal growth inhibition (PI) was calculated from the absorbance values of each treatment using the formula:

PI (%) = ((T1 − T2) − (T4 − T3))/(T1 − T2) × 100(1)

The MIC was defined as the lowest EO concentration capable of reducing fungal growth by approximately 95% [58].

The fungicidal effect of the EOs and BM was assessed by re-inoculating fungal spores onto Petri dishes containing solidified PDA medium, using 100 μL of samples that showed growth inhibition greater than 90%. Plates were incubated for 72 h at 25 °C under a 12 h photoperiod, and the MFC was defined as the lowest EO or BM concentration able to completely prevent fungal proliferation compared to the control [59].

Based on the PI results, the EC_50_ and its confidence intervals were calculated, representing the EO concentration that reduces fungal mycelial growth by 50% relative to the control.

### 4.3. Morphological Effect of Essential Oils from Eucalyptus staigeriana and Eucalyptus urograndis on the Fungus

The morphological alterations of *C. acutatum* caused by *E. staigeriana* and *E. urograndis* essential oils were analyzed using Scanning Electron Microscopy (SEM). A spore suspension (10^6^ spores mL^−1^) was cultured in potato broth at 25 °C. After fungal growth, the oils were added at concentrations of 2000 µL L^−1^ (*E. staigeriana*) and 4000 µL L^−1^ (*E. urograndis*), corresponding to the MICs determined in preliminary assays on solid medium (PDA). Control samples (without EO) were also included. The experiment was carried out in duplicates, with two independent replicates.

Following the protocol described by Escanferla et al. (2009) [60], the fungus was fixed, processed, dehydrated, critical-point dried, gold-coated, and examined using a LEO 435 Scanning Electron Microscope (Zeiss, Cambridge, UK). The most representative images were selected for morphological evaluation.

### 4.4. In Vivo Antifungal Activity of Essential Oils Incorporated into a Carboxymethylcellulose Edible Coating Against Colletotrichum acutatum

#### 4.4.1. Preparation of the Edible Coating

Five treatments were tested: control (C, no CMC or EO), preventive CMC (CMC_prev), preventive CMC + EO (CMC_EO_prev), curative CMC (CMC_cur), and curative CMC + EO (CMC_EO_cur).

Organic ‘Oso Grande’ strawberries collected in Cambuí (Minas Gerais, Brazil) were selected based on their ripeness stage and absence of visible damage. Subsequently, they were sanitized by immersion in a 2.5% chlorine solution for ten minutes.

The preparation of the CMC and its incorporation with the EO followed the methodology described by da Silva et al. (2020) [15]. In general, CMC (99.8% purity, pH 7.0, 340 cP viscosity, and degree of substitution 0.86) was used at 1%, dissolved in distilled water at 60 °C for 15 min under agitation (1000 rpm). Glycerol was then added at 0.5% as a plasticizer, with agitation for an additional 20 min. The essential oil of *E. staigeriana* was incorporated at a concentration of 6000 µL L^−1^ into the CMC solution. The oil was previously emulsified with Tween-80 (2:1, *v*/*v*) at 25 °C.

#### 4.4.2. In Vivo Antifungal Activity and Disease Evaluation

For the evaluation of the preventive mode of action, strawberries were immersed for 2 min in the edible CMC-based coating, with or without the addition of EO. After drying, fruits were inoculated with 30 μL of fungal suspension (10^5^ spores mL^−1^) applied onto a superficial wound approximately 3 mm deep. Subsequently, the fruits were maintained in a humid chamber (95% RH, 25 °C, 12 h photoperiod) for 24 h and then incubated for 7 days under the same conditions but outside the chamber.

For the curative mode of action, strawberries were first inoculated with the same fungal suspension (30 μL, 10^5^ spores mL^−1^) and kept for 24 h in the humid chamber to ensure optimal infection conditions. Only after this period was the CMC coating applied, with or without EO. The control treatment (C) was also inoculated and maintained under the same humid chamber conditions, but fruits received only sterile distilled water instead of the coating. Each treatment had 5 replicates consisting of 12 strawberries each. The experiment was conducted twice, with a one-year interval between trials.

Strawberries were assessed every 24 h over 7 days using a six-point disease severity scale based on the percentage of tissue affected by the fungus: 0 = no symptoms; 1 = 1–20%; 2 = 21–40%; 3 = 41–60%; 4 = 61–80%; and 5 = over 81%, following the method described by Oliveira et al. (2019) [39]. Severity data were converted into a disease index (DI%) using the formula from Cia et al. (2010) [61]:

DI (%) = {[(1 × n_1_) + (2 × n_2_) + (3 × n_3_) + (4 × n_4_) + (5 × n_5_)] × 100}/(5 × N) (2)

where n_i_ is the number of fruits in each score category, and N is the total number of fruits evaluated.

Based on the DI (%) values obtained for each treatment over time, the Area Under the Disease Progress Curve (AUDPC) was calculated using the method of Campbell and Madden (1990) [62]:

AUDPC = ∑\[(y_i_ + y_i_₊_1_)/2 × (t_i_₊_1_ − t_i_)] (3)

where y_i_ is the DI at time t_i_, and y_i_₊_1_ is the DI at the subsequent time t_i_₊_1_.

Additionally, disease incidence (%) was calculated as the proportion of symptomatic fruits at the end of the incubation period, based on the number of infected fruits on the last day of storage.

### 4.5. Evaluation of the Physicochemical and Sensory Quality of Strawberries Treated with CMC with Eucalyptus staigeriana EO

For these analyses, organic strawberries were also sourced from a local producer in Cambuí (Minas Gerais, Brazil). Fruits were visually selected based on uniform size, red coloration, overall appearance, and plant health. Sanitization was carried out by immersing the strawberries in a sodium hypochlorite solution (25 g L^−1^) for 10 min, followed by rinsing and air drying air-drying at room temperature (25 °C).

The fruits were then immersed for two minutes in one of the following treatments: sterile distilled water (Control, C), 1% carboxymethylcellulose (CMC), or 1% CMC coating incorporated with *E. staigeriana* essential oil at a concentration of 6000 µL L^−1^ (CMC_EO). The strawberries were packed in ventilated, transparent polyethylene clamshell containers (150 g capacity) and stored under refrigeration at 4.5 ± 0.5 °C and approximately 85% relative humidity for 15 days.

Analyses were conducted every three days, beginning on the day of processing (day 0) and continuing until day 15 of storage, totaling six evaluation points (0, 3, 6, 9, 12, and 15 days). Each treatment was performed in triplicate, with each replicate consisting of 150 g of fruit. The experimental design followed a completely randomized factorial scheme (3 × 6), with three treatments (C, CMC, and CMC + EO) and six storage periods.

#### 4.5.1. Physicochemical Parameters and Postharvest Quality

Weight loss (WL) was calculated as the percentage difference between the initial and final fruit weights. Pericarp disease incidence (PDI) was visually assessed and classified according to the surface area exhibiting symptoms of microbial growth, using a 0–3 scale [63]. Fungal spoilage (FS) was defined by the presence of visible mycelial growth on the fruit surface, and results were expressed as the percentage of infected fruits [64].

Pericarp coloration was measured using a colorimeter (Chroma Meter CR-400, Konica Minolta Sensing, Tokyo, Japan) with an 8 mm aperture and CIE standard illuminant C. The parameters evaluated included lightness (L), hue angle (in degrees), and chroma (color saturation).

Firmness (Fir) was determined with a digital penetrometer (Instrutherm, model PTR-300) equipped with a 3 mm probe, and results were expressed in Newtons (N).

For the determination of pH (method no. 981.12), total soluble solids (TSSs) (method no. 932.12, in °Brix), and titratable acidity (TA) (method no. 942.15, expressed as mg of citric acid per gram of fruit), the samples were homogenized in a mixer and filtered through gauze. The ratio was calculated as TSS/TA. All analyses were performed in triplicate, following the procedures described by AOAC (2005) [65].

To determine total monomeric anthocyanins (TMAs) and total phenolic compounds (TPCs), an extract was prepared from homogenized fruit using 80% acetone, as described by Petriccione et al. (2015) [66]. The extract was stored at 4 ± 1 °C for 24 h, centrifuged at 9500× *g* for 12 min at 4 °C, and the supernatant was frozen at −18 °C until analysis. Total monomeric anthocyanins were quantified by the pH differential method [65] and expressed as mg of cyanidin-3-glucoside equivalents per 100 g of fruit. Total phenolic content was determined using the method of Singleton and Rossi (1965) [67], with Folin–Ciocalteu reagent and sodium carbonate. Absorbance was measured at 740 nm using a spectrophotometer (JKI UVS-752N, Shanghai Jingke Scientific Instrument Co., Shanghai, China), and results were expressed as mg of gallic acid equivalents per gram of fruit.

#### 4.5.2. Sensory Analysis: Difference from Control Test

The sensory analysis was reviewed and approved by the Research Ethics Committee of ESALQ/USP (approval number: 55100316.8.0000.5395). The test was conducted in a single session, held after 6 days of refrigerated storage of the strawberries. The sensory panel consisted of 40 untrained participants of both sexes, 79% of whom were female, primarily aged between 18 and 25 years.

The Difference from Control Test [68] was used to determine whether there were significant differences (*p* ≤ 0.05) between the treatments (CMC and CMC_EO) and the control (C), as well as to estimate the magnitude of those differences. The test was conducted in individual sensory booths under white lighting, with strawberry samples served on white porcelain plates. Each panelist received a standard sample (*p*) and three coded samples (C, CMC, and CMC_EO), each labeled with a random three-digit code. The samples were presented to panelists in randomized block design. Panelists were instructed to compare the coded samples with the standard and to rate the degree of difference using an 11-point scale (0 = no difference from the standard sample; 10 = extremely different from the standard sample) [68]. Each panelist evaluated the samples from left to right, recording the corresponding score for the attributes aroma, color, and appearance.

The attributes evaluated were characteristic color, characteristic aroma, appearance, and overall sensory difference. Additionally, panelists were asked follow-up questions regarding their perceptions of the samples, including which sample they preferred and their opinions about the product’s aroma.

### 4.6. Statistical Analysis of the Results

The percentage values of fungal growth inhibition (PI) for *C. acutatum* were subjected to analysis of variance (ANOVA) using the F-test, and treatment means were compared by Tukey’s test at a 5% significance level. The EC_50_ for each essential oil, along with its 95% confidence interval, was determined via Probit analysis based on concentration–response log curves (logistic function), using PI (%) as the response variable. GenAI (OpenAI) was used to assist in generating a heat map representing the mean inhibition percentage (PI%) of *C. acutatum* growth at different concentrations (µL L^−1^) of *E. staigeriana, E. urograndis*, and their binary mixture.

The AUDPC results from the in vivo treatments against *C. acutatum* were also analyzed by ANOVA using the F-test, under a randomized block design corresponding to the two in vivo experiments. Treatment means were compared using Tukey’s test at a 5% significance level.

The physicochemical data of strawberries were subjected to Principal Component Analysis (PCA), a multivariate technique appropriate for evaluating complex datasets involving multiple variables simultaneously.

Sensory data were analyzed by ANOVA, followed by Dunnett’s multiple comparison test to compare the control sample with the treated groups (CMC and CMC_EO).

All statistical analyses were performed using the Statistical Analysis System (SAS), version 9.4.

## 5. Conclusions

This study successfully developed a biobased postharvest treatment for strawberries using *Eucalyptus staigeriana* essential oil, which showed strong antifungal activity against *C. acutatum*, outperforming *E. urograndis* and their binary mixture in vitro. Its superior efficacy was supported by morphological analysis and lower MIC, MFC, and EC_50_ values. In vivo, the curative application of the EO incorporated into carboxymethylcellulose significantly reduced anthracnose severity, confirming its potential as an effective postharvest control strategy. Treated fruits also exhibited reduced fungal spoilage during refrigerated storage. Despite some sensory changes, this study highlights the novelty of applying *E. staigeriana* EO in a CMC-based edible coating, offering a natural and promising alternative for managing postharvest fungal decay in strawberries. Further optimization is needed to improve sensory acceptance.

It is important to acknowledge certain limitations of this study, such as the use of a single fungal isolate and the absence of microbiological shelf-life evaluation, which may restrict the generalizability of the findings. Future studies should address these aspects and explore practical applications by optimizing EO formulations, testing under semi-commercial storage and distribution conditions, and assessing combinations with other biobased agents to enhance efficacy and sensory acceptance.

## Figures and Tables

**Table 1:** Mycelial growth inhibition (PI%), minimum inhibitory concentration (MIC), and minimum fungicidal concentration (MFC) of *C. acutatum* isolated from strawberry, after 48 h of exposure in microplate assays to essential oils (EOs) or binary mixture incorporated into liquid medium (mean values ± SD).

EO or Binary Mixture	Concentration (µL L^−1^)	PI (%)	MIC (µL L^−1^)	MFC (µL L^−1^)
*Eucalyptus staigeriana*	250	63.99 ± 8.49 B	>750	>2000
	750	98.53 ± 2.33 A		
	1000	99.44 ± 0.26 A		
	2000	97.92 ± 0.94 A		
	3000	98.48 ± 0.92 A		
	4000	97.72 ± 1.46 A		
	6000	99.40 ± 1.18 A		
	7000	97.93 ± 1.51 A		
*Eucalyptus urograndis*	250	52.71 ± 8.51 C	>6000	>7000
	750	70.44 ± 2.65 BC		
	1000	65.08 ± 9.21 C		
	2000	93.21 ± 4.76 A		
	3000	94.41 ± 6.85 A		
	4000	85.50 ± 5.25 AB		
	6000	100.0 ± 1.52 A		
	7000	97.41 ± 4.29 A		
Binary mixture	250	44.95 ± 4.52 C	>2000	>4000
	750	85.19 ± 2.92 B		
	1000	86.68 ± 10.13 B		
	2000	98.79 ± 1.47 AB		
	3000	100.0 ± 2.25 A		
	4000	99.10 ± 0.91 AB		
	6000	95.58 ± 2.95 AB		
	7000	94.80 ± 1.27 AB		

PI (%) = percentage of mycelial growth inhibition relative to the control treatment (1% DMSO solution with inoculum); SD = standard deviation of the mean. Different letters among EOs and the binary mixture indicate statistically significant differences at *p* < 0.05 according to Tukey’s test. Values represent the mean of three independent experiments, each with three replicates per treatment.

**Table 2:** Median effective concentration (EC_50_, μL L^−1^) and its 95% confidence intervals of essential oils and binary mixture against *C. acutatum* causing rot in strawberries. Mean of three experiments, each with three replicates per treatment.

EO or Binary Mixture	EC_50_ (µL L^−1^)	Confidence Intervals (95%)
*Eucalyptus staigeriana*	185.49	155.48–214.46
*Eucalyptus urograndis*	337.01	281.80–392.65
Binary mixture	355.62	314.70–396.31

**Table 3:** Area Under the Disease Progress Curve (AUDPC) for disease severity caused by *C. acutatum* in ‘Oso Grande’ strawberries subjected to treatments with or without *Eucalyptus staigeriana* essential oil (mean values ± SD, n = 60).

Treatments	AUDPC *
C	206.91 ± 41.46 AB
CMC_prev	182.14 ± 14.46 AB
CMC_EO_prev	223.13 ± 49.02 A
CMC_cur	168.49 ± 20.39 B
CMC_EO_cur	78.45 ± 11.59 C

Different letters on the columns indicate significant differences among treatments according to Tukey’s test (*p* < 0.05). SD = standard deviation of the mean; no. = number of fruits per treatment. C: untreated fruit (control); CMC_prev: fruit preventively treated with carboxymethylcellulose; CMC_EO_prev: fruit preventively treated with carboxymethylcellulose and essential oil; CMC_cur: fruit curatively treated with carboxymethylcellulose; CMC_EO_cur: fruit curatively treated with carboxymethylcellulose and essential oil. * The original data were transformed using the equation indicated by the Shapiro–Wilk test (ŷ = log_10_ y) and subjected to Tukey’s test for mean comparison.

**Table 4:** Results of physicochemical analyses of strawberries treated or not treated with CMC added with *Eucalyptus staigeriana* EO, stored under refrigeration for 15 days (mean ± standard deviation).

	Storage (Days)					
Treatments	0	3	6	9	12	15
	Weight Loss (%)					
C	0.00 ± 0.00	1.66 ± 0.75	3.07 ± 1.29	4.21 ± 1.70	5.73 ± 2.38	7.14 ± 2.24
CMC	0.00 ± 0.00	2.53 ± 0.68	5.56 ± 0.65	8.24 ± 1.30	11.07 ± 1.66	12.89 ± 1.59
CMC_EO	0.00 ± 0.00	2.62 ± 0.33	4.04 ± 0.82	5.74 ± 1.32	7.64 ±1.60	9.79 ±1.74
	Index of Diseases in the Pericarp					
C	0.00 ± 0.00	0.00 ± 0.00	4.44 ± 3.14	16.67 ± 5.44	43.33 ± 7.20	63.64 ± 4.29
CMC	0.00 ± 0.00	0.00 ± 0.00	4.44 ± 1.57	16.67 ± 2.72	44.44 ± 6.29	67.68 ± 5.63
CMC_EO	0.00 ± 0.00	0.00 ± 0.00	0.00 ± 0.00	4.44 ± 4.16	31.11 ± 17.50	55.35 ± 17.68
	Fungal Spoilage (%)					
C	0.00 ± 0.00	0.00 ± 0.00	16.36 ± 5.14	45.15 ± 12.25	81.90 ± 8.60	90.61 ± 0.43
CMC	0.00 ± 0.00	0.00 ± 0.00	10.00 ± 8.16	44.85 ± 10.66	65.15 ± 10.71	90.61 ± 7.44
CMC_EO	0.00 ± 0.00	0.00 ± 0.00	0.00 ± 0.00	12.42 ± 11.26	56.67 ± 26.25	83.64 ± 9.65
	Luminosity (L)					
C	36.75 ± 1.12	36.25 ± 0.35	36.10 ± 0.32	32.58 ± 0.60	34.80 ± 0.32	33.85 ± 0.98
CMC	35.83 ± 0.64	36.18 ± 1.16	36.11 ± 1.41	31.57 ± 1.34	34.19 ± 0.46	32.37 ± 1.64
CMC_EO	35.95 ± 0.54	35.10 ± 0.29	35.30 ± 0.09	33.12 ± 1.24	35.61 ± 0.67	32.60 ± 1.00
	Hue Angle (º)					
C	35.89 ± 1.79	36.33 ± 0.87	35.75 ± 0.33	38.07 ± 1.67	38.59 ± 1.11	38.24 ± 1.64
CMC	36.52 ± 0.66	39.36 ± 2.21	39.94 ± 2.51	38.59 ± 2.49	37.48 ±0.46	38.62 ± 1.18
CMC_EO	35.97 ± 1.14	33.49 ± 0.27	33.51 ± 0.33	34.26 ± 1.81	36.17 ± 0.65	38.36 ± 2.47
	Chromaticity					
C	40.06 ± 1.33	41.42 ± 0.23	41.40 ± 0.29	41.33 ± 0.66	43.24 ± 0.36	43.69 ± 1.50
CMC	42.67 ± 0.79	44.19 ± 0.64	44.07 ± 0.76	39.02 ± 0.18	41.48 ± 1.18	41.63 ± 2.35
CMC_EO	39.77 ± 0.47	36.64 ± 1.05	37.34 ± 0.44	35.86 ± 0.94	35.56 ± 1.31	36.80 ± 2.00
	Firmness (N)					
C	1.14 ± 0.19	1.38 ± 0.08	1.63 ± 0.24	1.57 ± 0.13	1.63 ± 0.05	1.46 ± 0.33
CMC	1.33 ± 0.13	1.50 ±0.16	1.43 ± 0.17	1.68 ± 0.16	1.37 ± 0.21	1.56 ± 0.05
CMC_EO	0.79 ± 0.14	0.68 ± 0.05	1.06 ± 0.15	0.96 ± 0.19	1.68 ± 0.46	1.40 ± 0.26
	pH					
C	3.69 ± 0.07	3.70 ± 0.04	3.63 ± 0.04	3.80 ± 0.05	3.67 ± 0.04	3.64 ± 0.07
CMC	3.73 ± 0.08	3.65 ± 0.04	3.77 ± 0.03	3.80 ± 0.05	3.83 ± 0.08	3.65 ± 0.02
CMC_EO	3.79 ± 0.05	3.72 ± 0.07	3.82 ± 0.03	3.87 ± 0.04	3.83 ± 0.03	3.84 ± 0.06
	Total Soluble Solids (Brix)					
C	8.22 ± 0.50	8.23 ± 0.14	7.82 ± 0.59	8.77 ± 0.69	8.20 ± 0.46	7.32 ± 0.18
CMC	8.37 ± 0.31	8.37 ± 0.10	8.20 ± 0.23	8.50 ± 0.23	8.68 ± 0.47	7.82 ± 0.66
CMC_EO	8.28 ± 0.05	8.12 ± 0.88	8.27 ± 1.09	7.92 ± 0.36	9.15 ± 1.76	7.78 ± 0.97
	Titratable Acidity (mg citric acid g^−1^ fruit)					
C	0.72 ± 0.03	0.63 ± 0.03	0.63 ± 0.02	0.74 ± 0.08	0.75 ± 0.02	0.72 ± 0.00
CMC	0.74 ± 0.02	0.62 ± 0.03	0.65 ± 0.00	0.67 ± 0.01	0.73 ± 0.03	0.76 ± 0.06
CMC_EO	0.74 ± 0.02	0.65 ± 0.00	0.66 ± 0.01	0.60 ± 0.04	0.68 ± 0.06	0.84 ± 0.07
	Ratio					
C	11.49 ± 1.21	13.15 ± 0.73	12.44 ± 0.89	11.95 ± 0.86	10.94 ± 0.27	10.20 ± 0.25
CMC	11.30 ± 0.53	13.56 ± 0.55	12.66 ± 0.43	12.65 ± 0.46	11.90 ± 0.53	10.32 ± 0.99
CMC_EO	12.53 ± 0.46	12.49 ± 1.37	12.48 ± 1.73	13.12 ± 0.28	13.40 ± 1.64	9.24 ± 0.46
	Total Monomeric Anthocyanins (mg cyanidin 3-glucoside equivalent 100 g^−1^ fruit)					
C	12.87 ± 0.26	20.21 ± 3.19	18.23 ± 3.36	21.94 ± 5.51	22.35 ± 1.76	16.55 ± 3.37
CMC	13.24 ± 0.02	16.98 ± 2.20	18.82 ± 1.70	20.85 ± 1.86	25.56 ± 2.64	17.69 ± 2.37
CMC_EO	14.11 ± 1.47	16.02 ± 3.83	17.32 ± 2.60	17.10 ± 2.44	12.78 ± 3.31	14.33 ± 0.52
	Total Phenolic Compounds (mg gallic acid equivalents g^−1^ fruit)					
C	0.356 ± 0.009	0.451 ± 0.044	0.452 ± 0.042	0.442 ± 0.015	0.528 ± 0.017	0.428 ± 0.007
CMC	0.412 ± 0.061	0.419 ± 0.015	0.424 ± 0.005	0.509 ± 0.024	0.554 ± 0.060	0.454 ± 0.055
CMC_EO	0.347 ± 0.025	0.368 ± 0.006	0.419 ± 0.010	0.435 ± 0.020	0.415 ± 0.028	0.530 ± 0.094

C = strawberries without treatment (control); CMC = strawberries treated with carboxymethyl cellulose; and CMC_EO = strawberries treated with carboxymethyl cellulose combined with essential oil of *Eucalyptus staigeriana*.

**Table 5:** Results of the Difference from Control Test for sensory attributes of strawberries treated with or without *E. staigeriana* essential oil and stored under refrigeration for 6 days.

	Sensory Attributes			
Treatments	Characteristic Color	Characteristic Aroma	Appearance	Overall Difference
C	1.88	3.00	3.13	2.85
CMC	3.08 *	4.90 *	4.05 n.s.	4.28 *
CMC_EO	5.55 *	7.53 *	6.38 *	6.95 *

C = control; CMC = strawberries treated with carboxymethylcellulose; CMC_EO = strawberries treated with CMC and *E. staigeriana* EO; * = significant difference (*p* < 0.05) between the sample and the control (C); and n.s. = no significant difference (*p* < 0.05) between the sample and the control.

## Data Availability

Data are contained within this article.
